# A Polarization-Independent Fiber-Optic SPR Sensor

**DOI:** 10.3390/s18103204

**Published:** 2018-09-22

**Authors:** Songquan Li, Laixu Gao, Changwei Zou, Wei Xie, Yong Wei, Canxin Tian, Zesong Wang, Feng Liang, Yanxiong Xiang, Qian Yang

**Affiliations:** 1 College of physical Science & Technology, Lingnan Normal University, Zhanjiang 524048, China; lisongquan1025@163.com (S.L.); gaolaixu@hlju.edu.cn (L.G.); changweizou@hotmail.com (C.Z.); xiewei@lingnan.edu.cn (W.X.); cxtian@lingnan.edu.cn (C.T.); zswang@lingnan.edu.cn (Z.W.); liangf@lingnan.edu.cn (F.L.); jxning@stumail.neu.edu.cn (Y.X.); 2 College of Electronic & Information Engineering, Chongqing Three Gorges University, Chongqing 404100, China; 20160009@sanxiau.edu.cn

**Keywords:** Fiber optics sensors, surface plasmon resonance, polarization-independent, bi-prism

## Abstract

Fiber-optic surface plasmon resonance (SPR) sensors possess the advantages of small size, flexible, allowing for a smaller sample volume, easy to be integrated, and high sensitivity. They have been intensively developed in recent decades. However, the polarizing nature of the surface plasmon waves (SPWs) always hinders the acquisition of SPR spectrum with high signal-noise ratio in wavelength modulation unless a polarizer is employed. The addition of polarizer complicates the system and reduces the degree of compactness. In this work, we propose and demonstrate a novel, polarization-independent fiber-optic SPR sensor based on a BK7 bi-prism with two incident planes orthogonal to each other. In the bi-prism, TM-polarized components of non-polarized incident lights excite SPWs on the first sensing channel, meanwhile the TE components and the remaining TM components are reflected, then the reflected TE components serve as TM components of incident lights for the second sensing channel to excite SPWs. Simulations show the proposed SPR structure permit us to completely eliminate the polarization dependence of the plasmon excitation. Experimental results agree well with the simulations. This kind of devices can be considered an excellent option for development of simple and compact SPR chemical sensors.

## 1. Introduction

Surface plasmon resonance is excited by an evanescent electromagnetic field produced in a attenuated total reflection structure. When the momentum of the incident light match that of surface plasmon wave, a sharp resonant dip occurs in the reflected light intensity. The propagation constant of the SPW strongly depends on the refractive index of the surrounding medium adjacent to the metal layer, therefore, SPR sensors have become among the most wide spread in their applications to disease diagnostics, food safety, environmental monitoring, and biomolecular interactions analysis in recent years [1,2]. Traditionally, the resonant condition is related to the incident angle or wavelength, and it is also critical for SPR to provide the right polarization. To excite SPW more effectively and obtain more obvious SPR phenomenon, transverse magnetic (TM)-polarized light is desired instead of transverse electric (TE)-polarized light due to SPW, which is TM-polarized and SPR phenomenon that is very polarization-sensitive.

Since Jorgenson and Yee proposed fiber-optic SPR sensor decades ago, many new types of fiber-optic SPR sensor have been increasingly developed possessing the advantages of small size, allows for a smaller sample volume, easy to be integrated [2,3]. Typically, flat metal layers are used in bulk optical Kretschmann configuration where polarizing devices are used to provide TM-polarized incident light with respect to the incident plane without ambiguity. When dealing with fiber-optic sensors, in the case of dual or multi-tapered, and side-polished structure [4,5]; TE and TM directions are well defined, in-line polarizers could be used prior to sensing region to extinguish TE polarization, but the polarization state of transmission light must be maintained until the sensing region, thus the fiber must be kept straight, without any torsion or stress to avoid birefringence, makes sensor complicated, and not easy to operate. For straight-tapered structure and uniform-waist tapered structure [6,7,8], one can no longer speak of TE and TM polarization since there is no well-defined incident plane. Some authors still use polarizing devices to maximize signal, but they refer these polarizations to a plane that cannot correspond to the sensing layers. A polarization-independent SPR fiber sensor without any polarizer has been demonstrated [9], but the uniform-waist tapered structure suffers from the disadvantage of poor mechanical stability, similar to other sensors using optical fiber for coupling. From a practical point of view, the fiber-optic SPR sensor whose coupling device is optical fiber is fragile, in addition, it needs to be fabricated using specialized equipment that is usually expensive.

Fiber-optic SPR sensors with coupling prism and fibers that only transmit SPR signals have been proposed years ago [10,11]. Such sensors, compared with conventional fiber-optic SPR sensors, are more robust since the coupling prism is not as fragile as bare fiber. Besides, they can have a wide and selectable detection range that depends on the selected material and physical dimension of prism. By selecting the refractive index of the prism or the adjusting incident angle, detection range and sensitivity can be tuned conveniently. However, these sensors are commonly spectrally interrogated, so it is necessary to use polarizing elements to provide TM-polarized light for higher signal-noise ratio, which would complicate the setup and reduce the degree of compactness. Recently, a compact fiber-optic SPR sensor operating in the telecommunications C-band has been reported [12]. The sensor is designed for intensity modulation, thus the absence of polarizer does not affect the relative change of intensity signal. Nevertheless, the fluctuation of light intensity and variation of polarization state will deteriorate the accuracy and repeatability of measurement results unless necessary techniques, such as differential operation or phase-locked amplification, are employed.

In this paper, we propose a novel polarization-independent fiber-optic SPR sensor based on a BK7 bi-prism with two incident planes orthogonal to each other. In view of drawbacks of intensity modulation, the proposed sensor is designed for wavelength interrogation. In the bi-prism, TM-polarized components of non-polarized incident lights excite SPWs on the first sensing channel, meanwhile the TE components and the remaining TM components are reflected, then the reflected TE components serve as TM components of incident lights for the second sensing channel to excite SPWs. Consequently, the non-polarized incident lights are sufficiently utilized without any additional polarizer. To the best of our knowledge, no device of such configuration has been reported in the literature, and it is important to note how convenient and effective such sensing structure will be for the development of more robust and compact fiber-optic SPR sensors.

## 2. Sensor Structure

**Figure 1:**
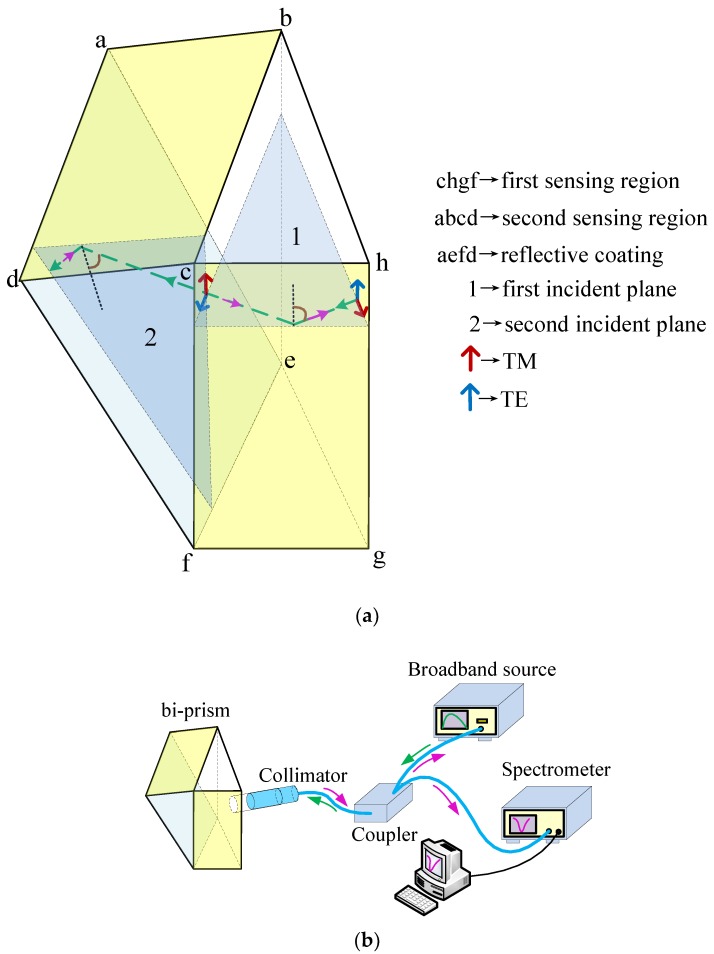
(**a**) Proposed sensor based on bi-prism. The bi-prism reverses the polarization state of light reflected by first sensing region. The reflected light strikes the second sensing region, then be reflected by the reflective coating normally; and (**b**) Schematic diagram of sensing system.

BK7 glass is very popular in SPR technique, also widely used in optics resulting in low customization costs. The custom-made BK7 prisms have isosceles-triangular base of angles 74.65°, corresponding to the cross section dimensions 34 mm × 34 mm × 18 mm and height 34 mm. Figure 1a shows the proposed sensor structure. The bi-prism comprises two BK7 prisms that are bonded together with UV curing glue in the form of two incident planes orthogonal to each other.

Au film is selected as a sensing layer owing to its good chemical stability. Sensing layer (70 nm) was deposited on two sensing region simultaneously to maintain consistency. Thick Ag film capped with SiO_2_ film for protection and preventing oxidation is used as reflective coating. Ag film (~500 nm) was deposited onto square facet of prism followed by SiO_2_ film (~500 nm) using magnetron sputtering apparatus (FJL560CⅡ, Sky Technology Development Co., Ltd., Shenyang, China). Prisms were cleaned with acetone before nitrogen dry, and then bonding process was carried out using UV curing glue and UV lamp. A multi-mode fiber collimator with FC/PC connector (diameter of 62.5 μm) was inserted into the rigid plastic pipe and fixed with UV glue followed by UV reinforcement. Bi-prism and collimator are mounted on 3-D adjustment devices, respectively. After alignment, the pipe was carefully glued on the entrance side of bi-prism as depicted in Figure 1b. The sensor is ready to be tested when the final bonding process was completed.

The sensor presented here is spectrally interrogated; a reasonable broadband source should be employed. Light from tungsten halogen lamp (HLS-1, KEWLAB Pty Ltd., Melbourne, Australia) is launched into one port of the multi-mode fiber coupler (diameter of 62.5 μm) and route to the collimator. Without apolarizer, the incident light emerging from collimator can be considered to be non-polarized light which then strikes the first sensing region at an angle of 74.65°. When the bi-prism is immersed to an aqueous solution of appropriate refractive index within the detection range, the TM components of incident light produce an evanescent field that excites SPW at a certain wavelength, while TE components are intensively reflected after undergoing minor intrinsic loss caused by Au film. Due to two incident planes of prism are mutually orthogonal, the unique bi-prism structure reverses the polarization state of light which was reflected by first sensing the region, as Figure 1a depicted, consequently, the original TE components transform to TM components which then strikes the second sensing region at an angle of 74.65° and excites SPW. After normal reflection back off the reflective coating, the light strikes the two sensing regions again at the same incident angle, and eventually couples back through the collimator into the fiber coupler routing half part of optical signal to an optic spectrometer (USB4000, Ocean Optics, Inc., Dunedin, FL, USA), from which SPR spectrum of wavelength modulation can be analyzed.

In the proposed SPR sensor configuration, the incident light emerging from collimator interacts twice with SPW for both TM and TE components, so multilayer Transfer Matrix Method for both TM and TE-polarized light should be considered. The following equation represents the reflectivity $R_{\gamma }$ where γ refers to TM or TE.

(1) $$R_{\gamma }=\frac{r_{prm}+r_{ms}\exp\left(2ik_{zm}d\right)}{1+r_{prm}r_{ms}\exp\left(2ik_{zm}d\right)}\;$$

For TM-polarized light,

(2) $$r_{prm}=\frac{\varepsilon_{m}k_{zpr}-\varepsilon_{pr}k_{zm}}{\varepsilon_{m}k_{zpr}+\varepsilon_{pr}k_{zm}}\;$$

(3) $$r_{ms}=\frac{\varepsilon_{s}k_{zm}-\varepsilon_{m}k_{zs}}{\varepsilon_{s}k_{zm}+\varepsilon_{m}k_{zs}}\;$$

For TE-polarized light,

(4) $$r_{prm}=\frac{k_{zpr}-k_{zm}}{k_{zpr}+k_{zm}}\;$$

(5) $$r_{ms}=\frac{k_{zm}-k_{zs}}{k_{zm}+k_{zs}}\;$$

$r_{prm}$ and $r_{ms}$ are the reflection coefficients for prism-Au interface and Au-sample interface, respectively. $k_{zj}$ is the wave vector perpendicular to the interface in the medium j (*pr* for prism, *m* for metal film and *s* for sample). $\varepsilon_{j}$ is the dielectric constant of medium j. d is the Au film thickness. $k_{x}$ is the wave vector parallel to the interface in prism.

(6) $$k_{zj}=\sqrt{\varepsilon_{j}{\left(\frac{\omega }{c}\right)}^{2}-k_{x}^{2}}\;$$

(7) $$k_{x}=\sqrt{\varepsilon_{pr}}\left(\frac{\omega }{c}\right)\sin\theta_{pr}\;$$

**Figure 2:**
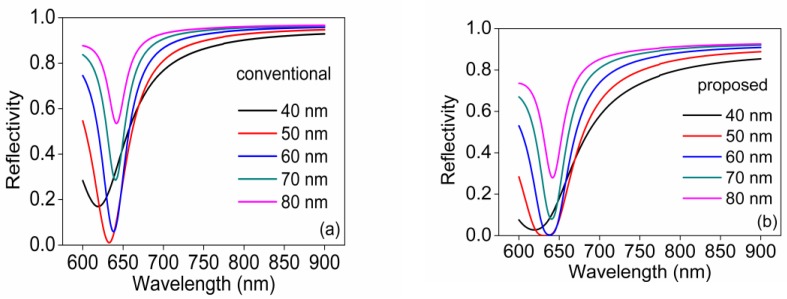
SPR spectrum for sample refractive index of 1.33, and Au film thickness of 40 nm, 50 nm, 60 nm, 70 nm and 80 nm. (**a**) The conventional sensor based on single BK7 prism; and (**b**) The proposed sensor based on bi-prism.

Since the two reflections on the sensing regions are identical, the reflectivity of bi-prism $R_{total}$ can be expressed as $R_{total}={\left(R_{TM}\right)}^{2}\cdot {\left(R_{TE}\right)}^{2}$. To compare the performances of the proposed sensor and conventional Kretschmann configuration, we carried out numerical simulations. In simulation, we took the dielectric constants of Au from Reference [13]. Figure 2 shows the calculated SPR spectrum for sample refractive index of 1.34, and Au film thickness of 40 nm, 50 nm, 60 nm, 70 nm, and 80 nm. From Figure 2, we observed that the resonant dip shallows as well as the full width at half maximum (FWHM) narrows as the film thickness increases for both sensing structure. Moreover, at the same thickness of Au film, the reflectivity of proposed sensor is smaller and the FWHM is wider than that of conventional sensor attributes to the $R_{TE}$ is not 100% and the double-pass of light path originated by bi-prism as well. It can clearly be seen that the bottom of resonant dip flattens in the case of 50 nm Au film for the proposed sensor which is detrimental to distinguish the resonant wavelength. As a result, considering the depth and FWHM of SPR spectrum, the Au film thicknesses from 60 nm to 70 nm are suitable; we chose 70 nm Au film as a sensing layer for wavelength modulation to avoid flat SPR dip. What should be mentioned is the proposed sensor based on bi-prism can provide a good enough SPR spectrum for sensing without the aid of polarizer according to the simulation results.

## 3. Experiments and Discussion

**Figure 3:**
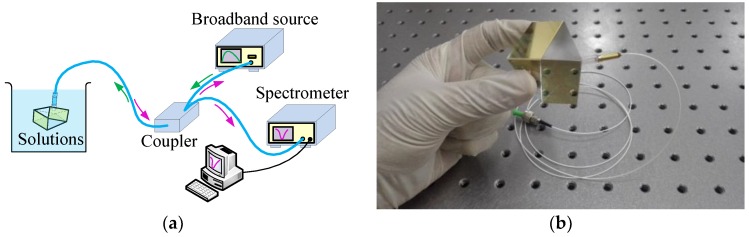
(**a**) Experimental setup for detect the refractive index of aqueous solution; and (**b**) Photograph of sensor based on bi-prism.

Figure 3a depicts the proposed sensing system for detecting the refractive index of aqueous solution, which aims to demonstrate the concept of the proposed sensor. The sensor based on bi-prism is illustrated in Figure 3b.

**Figure 4:**
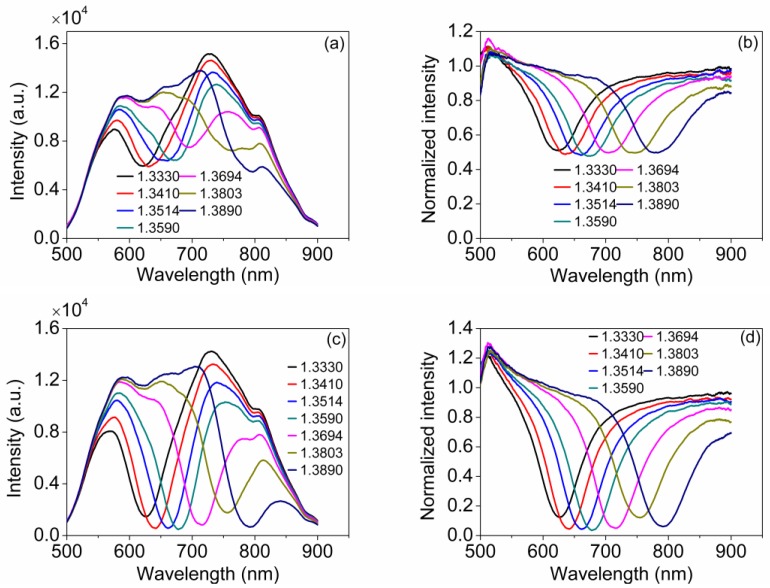
(**a**) Testing results of the spectrum reflected back off the sensor when only the second sensing region is immersed into glycerine aqueous solutions; (**b**) Corresponding normalized SPR spectrums; (**c**) Testing results when the whole bi-prism is immersed into glycerine aqueous solutions; and (**d**) Corresponding normalized SPR spectrums.

In the experiment, glycerine aqueous solutions are sampled for refractive index detection, in which the bi-prism is immersed, as Figure 3a illustrated. The index is measured and calibrated by the Abbe refractometer with a resolution of 10^−4^. A spectrometer measures the spectrum reflected back off the sensor. Before the sensor was immersed in liquid, we measured a spectrum and took it as the reference spectrum for normalization. Figure 4a shows the testing results of the spectrum reflected back off the sensor in the glycerine aqueous solutions with indexes of 1.3330, 1.3410, 1.3514, 1.3590, 1.3694, 1.3803, and 1.3890 when only the second sensing region is immersed into glycerine aqueous solutions. The normalized SPR spectrums are shown in Figure 4b, from which it can be seen clearly that the SPR dips are shallow and the minimum reflectivity’s are about 0.5. The phenomenon indicates that nearly all of the TE-components of the incident light with respect to the first sensing region at these SPR wavelengths were absorbed while the TM-components were basically remained. Besides, it is also indicated that the incident light is nearly a non-polarized light.

Figure 4c shows the testing results of the spectrum reflected back off the sensor in the glycerine aqueous solutions when the whole bi-prism is immersed into glycerine aqueous solutions. The normalized SPR spectrums are shown in Figure 4d, from which it is obvious that the proposed sensor is capable of providing good SPR responses with the absence of a polarizer as expected in simulations. The reflectivity’s at resonant wavelengths agree well with those from simulations. There is some discrepancy between simulations and measurements caused by several reasons: For simplicity of simulations, we ignored the difference of incident angles against the film corresponding to different wavelengths caused by the dispersion effect of the prism and the divergence of collimated output beam, which will broaden experimental SPR spectrum. In addition, the reference spectrum contains the absorption factor of Au and Ag films, and is not synchronously measured, which have an impact on the reflectivity at resonant wavelength. All these factors contribute to the discrepancy between simulations and measurements.

**Figure 5:**
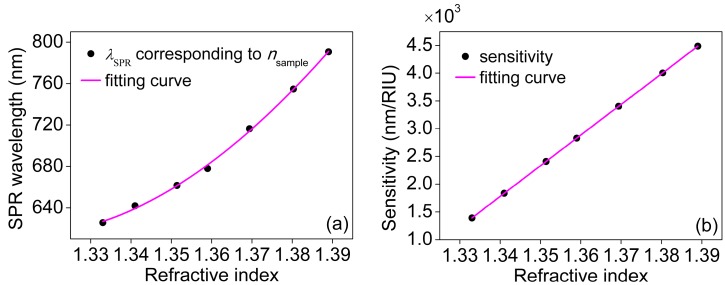
(**a**) Relation between the refractive index and the resonant wavelength; and (**b**) Relation between the refractive index and the refractive index dependent sensitivity.

Resonant wavelengths are extracted from the normalized SPR spectrums from Figure 4d and Figure 5a show the relationship between the refractive index of sample and the resonant wavelength. The slope of the curve equals to the refractive index dependent sensitivity. Figure 5b provides the relation between the refractive index of sample and the refractive index dependent sensitivity. Figure 5 indicates that the sensitivity and the resonant wavelength increase as the refractive index increases, which are in accordance with the conclusions in the literature [2]. According to the testing results, the maximum sensitivity is up to 4392 nm/RIU.

The signal to noise ratio (SNR) of SPR sensor depends on how accurately and precisely the sensor can detect the resonant wavelength. The resolution associated with SNR and the full width at half maximum (FWHM) of SPR spectrum is a proper parameter to evaluate the proposed sensor. As a result, we calculated the SNR of each normalized SPR spectrum by analyzing the experimental results. In analysis, spectral data below half maximum were adopted, and each SNR was calculated using the formula in the literature [14], which is expressed as $SNR=1/\sigma^{2}$, where $\sigma^{2}$ is the variance of amplitude noise. The resolution limits of resonant wavelengths were derived from the following equation, which is also sourced from Reference [14].

(8) $$\Delta \nu_{SPR}=\frac{2\sqrt{2}}{\pi }\sqrt{\frac{dv\cdot \Delta \nu }{SNR}}\;$$

In the equation, $\Delta \nu_{SPR}$ is resolution limit of resonant wavelength in frequency domain, dv is spectral resolution at resonant wavelength in frequency domain, Δν is linewidth of FWHM in frequency domain. We traded $\Delta \nu_{SPR}$ with spectral resolution. As shown in Table 1, it is obvious that the resolution limits of resonant wavelengths are worse than the resolution of 0.22 nm provided by the spectrometer, which is attributed to the influence of spectral amplitude noise and FWHM of SPR spectrum on the detection accuracy of the resonant wavelength. The resolution of the proposed sensor is dependent on the refractive index of sample and is improved with the increase of refractive index. The highest resolution of the proposed sensing system is $2.26\times 10^{-4}$ RIU. Higher resolution can be expected if the spectrum of the light source is sufficiently stable and smooth as well as a spectrometer with higher optical resolution is employed. While the performances of proposed sensor are not as better as those of sensors with coupling devices of a lower refractive index (such as fiber), the experimental results can still demonstrate the proposed sensor based on bi-prism is capable of providing good SPR responses without the aid of polarizer.

**Figure 6:**
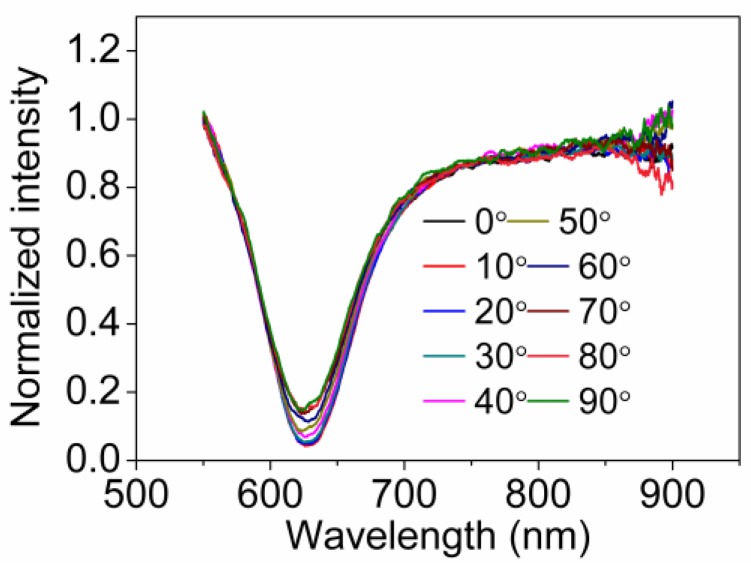
Normalized SPR spectrums at each polarization azimuth.

To prove the property of polarization independent possessed by the bi-prism, we removed the collimator from bi-prism and cleaned up the UV curing glue on the entrance side of light with acetone. The bi-prism is amounted on a 2D adjusting frame. We chose a collimator with a working distance of 5.9 mm and a clear aperture of 4.5 mm (CFC-8X-B, Thorlabs, Inc., Newton, NJ, USA) instead of the collimator with working distance of 2 mm and clear aperture of 1.8 mm we used before. A Glan-Taylor polarizer (GCL-070211, Daheng New Epoch Technology, Inc., Beijing, China) is used to adjust the polarization direction of light output by the collimator. Light from tungsten halogen lamp is launched into one port of the multi-mode fiber coupler and route to the collimator. The incident light emerging from collimator then enters the bi-prism. After normal reflection back off the reflective coating, the light strikes the two sensing regions again, and couples back through the collimator into the fiber coupler routing half part of optical signal to the optic spectrometer. Due to the limitation of the experimental structure, the prism can hardly be immersed into liquid. Therefore, in the experiment, the water droplets were dripped onto two sensing regions for SPR measurement. The polarization direction of light with respect to the first incident plane (here we call it polarization azimuth) was adjusted at intervals of 10° from 0° to 90° by rotating the polarizer axially. To reduce the impact of alignment errors and degree of polarization of light output by the collimator on the experimental results, we collected reference spectrums for normalization at each polarization azimuth previously. The normalized SPR spectrums at each polarization azimuth are shown in Figure 6.

From Figure 6, it can be observed that the variation of resonant wavelength is minor, which indicates the incident angles corresponding to two sensing regions are basically identical. However, the reflectivity at resonant wavelength increases as the polarization azimuth increases, also the normalized SPR spectrums became shallow. The main reason is that the beam output by the collimator has certain divergence, and consequentially, the spot area in the second sensing region is larger than the spot area in the first sensing region, thus, the intensity of light which strikes the second sensing region with resonant angle is lower, as is the intensity at the resonance wavelength. When the polarization azimuth is 0°, the second sensing region only serves as a reflecting mirror while the first sensing region is activated, in this case, the intensity of the light that excites SPR is higher, and the intensity of the light returned to the spectrometer is lower. When the polarization azimuth is 90°, the first sensing region only serves as a reflecting mirror while the second sensing region is activated, the intensity of the light that excites SPR is lower, and the intensity of the light returned to the spectrometer is higher. Therefore, the reflectivity at resonant wavelength increases as the polarization azimuth increases. The proposed sensor ideally should be polarization independent. However, owing to the existence of beam divergence, the spectrums in Figure 6 cannot be completely coincided. The improvement of the coincidence of the spectrums can be expected when the spot area, prism and beam divergence is small enough. Nevertheless, the experimental results prove that it is feasible to use the proposed sensor based on bi-prism to eliminate polarization dependence of SPR excitation.

Compared to other fiber-optic SPR sensor, the major benefits of the proposed one are as follows: First of all, it works most without any polarizing element providing deep enough SPR dip for wavelength modulation, which makes it cheaper. The prism can be customized to a smaller size to form a more compact structure. Second, unlike the sensor whose coupling device is optical fiber, the proposed sensor uses prism as a coupling element avoiding the risk of fragility of the coupling elements, which makes it more robust and more convenient to operate. Third, conventional fiber-optic SPR sensors typically have a limited detection range due to the refractive index of coupling fiber, while the proposed sensor has a wide detection range that depends on the selected material of a prism with a larger refractive index than that of fiber. Fourth, the SPR condition is tunable since thickness of dielectric overlayer on Au film can affect the SPR angle or wavelength, thus detection range and sensitivity can be tuned conveniently. Fifth and the last, a wider detection range can be achieved by employing a spectrometer with a wider detection range of wavelengths. The prism also can be customized to detect target gas under the coordination of functional membranes on the SPR-compatible layer. When the proposed sensor works in the mode of intensity interrogation, which is not the work this paper involves, the bi-prism structure can greatly reduce the dependence of the measurement accuracy and repeatability on the polarization state of the light source, which can improve the adaptability of the sensor to light sources making the sensor more practical.

## 4. Conclusions

In this paper, we present a polarization-independent fiber-optic SPR sensor based on bi-prism with two incident planes perpendicular to each other. On the basis of the polarizing nature of the SPW and the method to stimulate the SPW, we show how the polarization-dependence of SPR disappears when the bi-prism is employed. From the practical point of view, the sensor is very simple and effective. Furthermore, the unique properties of the proposed SPR sensor are especially beneficial to various emerging sensing applications, such as toxic gas detection, safety analysis, and environmental monitoring. Not only the sensor can be operated in wavelength interrogation, but also in intensity interrogation, especially in the optical fiber communication band for remote online monitoring. The related development is ongoing.

## Figures and Tables

**Table 1:** Comparison of sensor parameters corresponding to refractive index of seven samples.

Parameters	1.3330	1.3410	1.3514	1.3590	1.3694	1.3803	1.3890
*λ_SPR_*, nm	625.67	641.89	661.54	677.93	716.35	754.73	790.62
Sensitivity, nm/RIU	1315	1807	2424	2856	3422	3982	4392
FWHM, nm	100.00	104.56	108.17	114.77	129.48	136.52	159.23
SNR, dB	30.39	30.18	29.75	30.40	26.44	29.51	26.65
RL of *λ_SPR_*^1^, nm	0.76	0.78	0.80	0.81	0.92	0.90	1.01
Res^2^, $\times 10^{-4}$ RIU	5.83	4.34	3.30	2.85	2.71	2.26	2.30

^1^ RL of λ_SPR_ refers to the resolution limit of resonant wavelength; ^2^ Res refers to the resolution of the sensing system.
